# Interactions between Casein Kinase Iε (CKIε) and Two Substrates from Disparate Signaling Pathways Reveal Mechanisms for Substrate-Kinase Specificity

**DOI:** 10.1371/journal.pone.0004766

**Published:** 2009-03-10

**Authors:** Caroline Lund Dahlberg, Elizabeth Z. Nguyen, David Goodlett, David Kimelman

**Affiliations:** 1 Department of Biochemistry, University of Washington, Seattle, Washington, United States of America; 2 Department of Medicinal Chemistry, University of Washington, Seattle, Washington, United States of America; University of Washington, United States of America

## Abstract

**Background:**

Members of the Casein Kinase I (CKI) family of serine/threonine kinases regulate diverse biological pathways. The seven mammalian CKI isoforms contain a highly conserved kinase domain and divergent amino- and carboxy-termini. Although they share a preferred target recognition sequence and have overlapping expression patterns, individual isoforms often have specific substrates. In an effort to determine how substrates recognize differences between CKI isoforms, we have examined the interaction between CKIε and two substrates from different signaling pathways.

**Methodology/Principal Findings:**

CKIε, but not CKIα, binds to and phosphorylates two proteins: Period, a transcriptional regulator of the circadian rhythms pathway, and Disheveled, an activator of the planar cell polarity pathway. We use GST-pull-down assays data to show that two key residues in CKIα's kinase domain prevent Disheveled and Period from binding. We also show that the unique C-terminus of CKIε does not determine Dishevelled's and Period's preference for CKIε nor is it essential for binding, but instead plays an auxillary role in stabilizing the interactions of CKIε with its substrates. We demonstrate that autophosphorylation of CKIε's C-terminal tail prevents substrate binding, and use mass spectrometry and chemical crosslinking to reveal how a phosphorylation-dependent interaction between the C-terminal tail and the kinase domain prevents substrate phosphorylation and binding.

**Conclusions/Significance:**

The biochemical interactions between CKIε and Disheveled, Period, and its own C-terminus lead to models that explain CKIε's specificity and regulation.

## Introduction

CKI family members have been implicated in a wide range of signaling activities including involvement in the Wnt, planar cell polarity (PCP) and circadian rhythms pathways [1]–[3]. In mammals, the CKI family of serine/threonine kinases consists of seven distinct genes (α, β, γ_1_, γ_2_, γ_3_, δ, ε) that code for enzymes with highly conserved kinase domains and divergent amino- (N-) and carboxy- (C-) termini. CKIε in particular has a long (∼100 residue) C-terminus that undergoes inhibitory autophosphorylation at conserved serine and threonine residues [4]–[7]. Subcellular localization of different isoforms in some cases is determined by splice variants (in the case of CKIα) [8], [9] or fatty-acylation (in the case of CKIγ) [10], but Western and Northern blotting experiments, and in situ hybridization studies indicate that the α, δ, and ε isoforms have generally overlapping expression [9], [11]–[14]. It is therefore interesting that despite high sequence similarity and common expression patterns, CKI family members have different targets in the cell [10], [15]–[18].

Dishevelled (Dsh) is a 100 kDa protein that is required for canonical Wnt and Frizzled/PCP signaling [19], [20]–[23]. Hyperphosphorylation is accepted as an indicator of Dsh's activation in both pathways [18]–[30]. During canonical Wnt signaling, phosphorylated Dsh induces the translocation of Wnt responsive signalosomes to the cell membrane, as an early step in stabilizing the transcriptional coactivator, β-catenin [26]–[31]. Dsh's phosphorylation also indicates its activation in Frizzled/PCP signaling [2], [19], [21]–[33]. Although CKIα, CKIγ, and CKIε have all been implicated in Wnt and/or Frizzled/PCP signaling, only CKIε has been shown to bind to and phosphorylate Dsh, in vivo. Despite the similarity between the kinase domains of CKIε and the other CKI isoforms, CKIε is the kinase required for the full activation of Dsh and its downstream effects [19], [23], [27].

Physiological outputs (hormonal, metabolic and cellular) that are entrained to the 24 hour cycle of the earth's rotation have been described in organisms from cyanobacteria through humans, and are based on molecular clockwork, [reviewed in 3], [34], [35]. The cyclic expression and localization of the transcriptional regulator, Period (Per), is in large part regulated by phosphorylation by CKIε in mammals [36]–[40]. There are three Per isoforms in mice (mPer1, mPer2, mPer3) that display different expression and regulation patterns [41], [42]. Mutations in CKIε disrupt the circadian pathway through aberrant or reduced phosphorylation of Per [39], [43]–[45]. Both CKIε and CKIα isoforms are found in cells that exhibit circadian cycling, including the master circadian oscillator in mice, the suprachiasmatic nucleus [14]. Why one CKI isoform is active in the pathway while the other is not has not been investigated.

Per and Dsh bind specifically to CKIε [19], [37], [46]–[48]. To determine how CKIε and CKIα differ with respect to binding by substrates, we performed a series of in vitro experiments to probe the direct interaction between CKIε and two substrates from disparate signaling pathways. We show that, surprisingly, features C-terminal to the kinase domain of CKIε are not responsible for the difference in binding of the substrate proteins to the two CKI kinases. Instead, two residues on the kinase domain of CKIε determine Dsh's and Per's affinity for CKIε, whereas the C-terminus of CKIε stabilizes interactions between CKIε and Dsh and Per. Finally, we show that autophosphorylation of CKIε's C-terminus inhibits substrate binding. Using chemical cross-linking and tandem mass spectrometry, we demonstrate that autophosphoryation of the C-terminal tail changes its interaction with the kinase domain, revealing why the autophosphorylated tail inhibits substrate phosphorylation and binding.

## Results

### CKIε, but not CKIα, binds to xDsh and mPer1 in vitro

**Figure 1:**
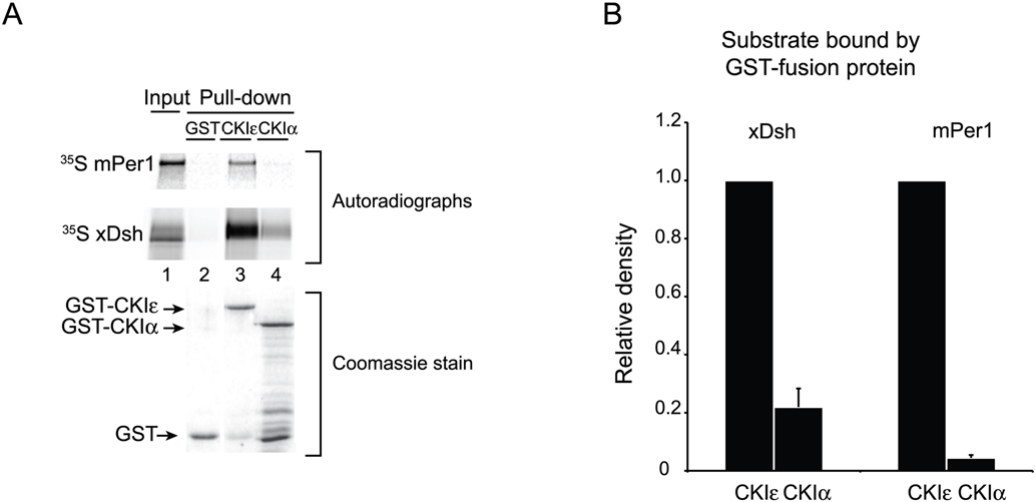
Recombinant CKIε, but not CKIα, interacts with mPer1 and xDsh. (A) GST, GST-CKIε, or GST-CKIα was bound to glutathione sepharose, and then incubated with ^35^S-labeled mPer1 or xDsh. 10% of the mPer1 input and 25% of the xDsh input are shown. Coomassie stained gel shows the levels of GST fusion protein used for each pull-down. (B) Quantification of three independent experiments. Values are normalized against the amount of protein bound by GST-CKIε, and error bars represent standard deviation of the mean.

Per1 and Dsh bind to CKIε in vivo, but they do not interact with the related isoform CKIα [23], [47]–[49]. In order to determine if these substrates bind to the two kinases with the corresponding affinities in vitro, we used a Glutathione S-Transferase (GST) pull-down system to follow protein-protein interactions. Due to protein instability and toxicity in *E. coli*, prior in vitro studies of CKIε have used a C-terminally truncated form of the protein called CKIεΔC [48], [50], [51], which eliminates the last 98 residues of the protein. Since we wanted to include all of the protein in our analysis, we developed an expression system for large quantities of pure full-length *Xenopus* CKIε fused to GST (CKIε-GST; see Methods). Using a similar strategy we also expressed full-length *Xenopus* CKIα fused to GST (CKIα-GST). Both kinases were catalytically active against β-catenin, indicating that they were both well folded (data not shown). Because CKIε autophosphorylation regulates substrate binding (see below), we used dephosphorylated CKIε for our binding studies except where otherwise noted. Using GST-pull-down assays we found that ^35^S-labelled *Xenopus* Dsh (xDsh) and mouse Per1 (mPer1) bound specifically to CKIε compared to CKIα (Fig. 1), recapitulating previously published in vivo results [19], [37], [46]–[48]. This provided us with a useful assay system to examine CKIε specificity for these substrates.

### CKIε does not require its C-terminus to interact with substrate proteins

**Figure 2:**
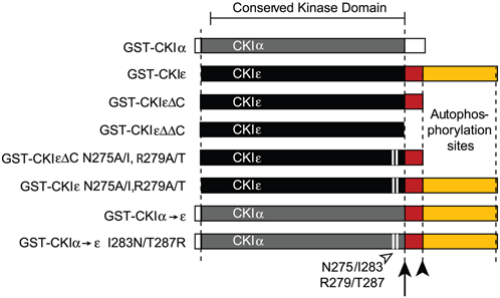
Protein constructs used in this work. CKIα and -ε are shown with their conserved kinase domains in gray and black, respectively (89% similarity, 75% identity). The arrow indicates the position of residue 295 in CKIε, and the non-conserved, charged region of the protein is colored red. The filled arrowhead indicates the position of residue 319, where CKIε is conventionally truncated. The C-terminus contains autophosphorylation sites and is colored yellow. The open arrowhead and white bars indicate the position of residues N275 and R279 (CKIε), and I283 and T287 (CKIα).

**Figure 3:**
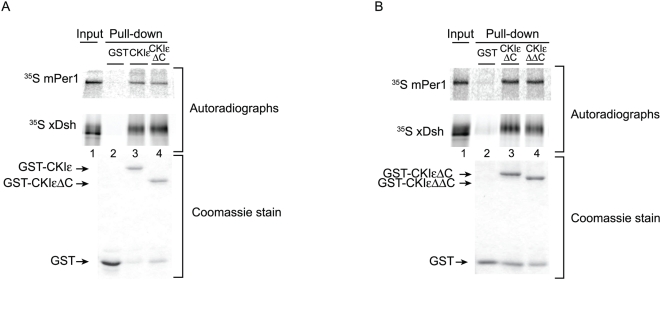
xDsh and mPer1 do not require CKIε's C-terminus for binding. (A, B) Purified GST, GST-CKIε, GST-CKIεΔC or GST-CKIεΔΔC were bound to glutathione sepharose and incubated with ^35^S-labeled xDsh or mPer1 as indicated.

CKIε and CKIα are 75% identical, and 89% similar in their kinase domains (Fig. 2). This high degree of conservation between the kinases suggested that full binding by mPer1 and xDsh to CKIε might depend on the long C-terminal tail, which is unique to CKIε. We first examined binding of mPer1 and xDsh to CKIεΔC, which truncates the kinase at residue 319 (Fig. 2). Figure 3A shows that xDsh and mPer bound to CKIε and CKIεΔC to the same extent, demonstrating that CKIε does not require the autophosphorylation region of the C-terminus to bind to substrate proteins.

Because truncation of CKIε at residue 319 did not diminish xDsh or mPer1's affinity for the kinase, another part of the protein must determine CKIε's interaction with substrate proteins. CKIεΔC retains 22 residues that are not conserved between CKIε and CKIα (Fig. 2) and these could contribute to the substrate-kinase interaction. We produced CKIεΔΔC, which is truncated at residue 295 and therefore even more closely resembles CKIα (Fig. 2). Figure 3B shows that CKIεΔC and CKIεΔΔC each bound to xDsh or mPer1with the same affinity. CKIε must therefore bind to substrate proteins through its kinase domain.

### Residues N275 and R295 are responsible for enhanced substrate binding to CKIε

**Figure 4:**
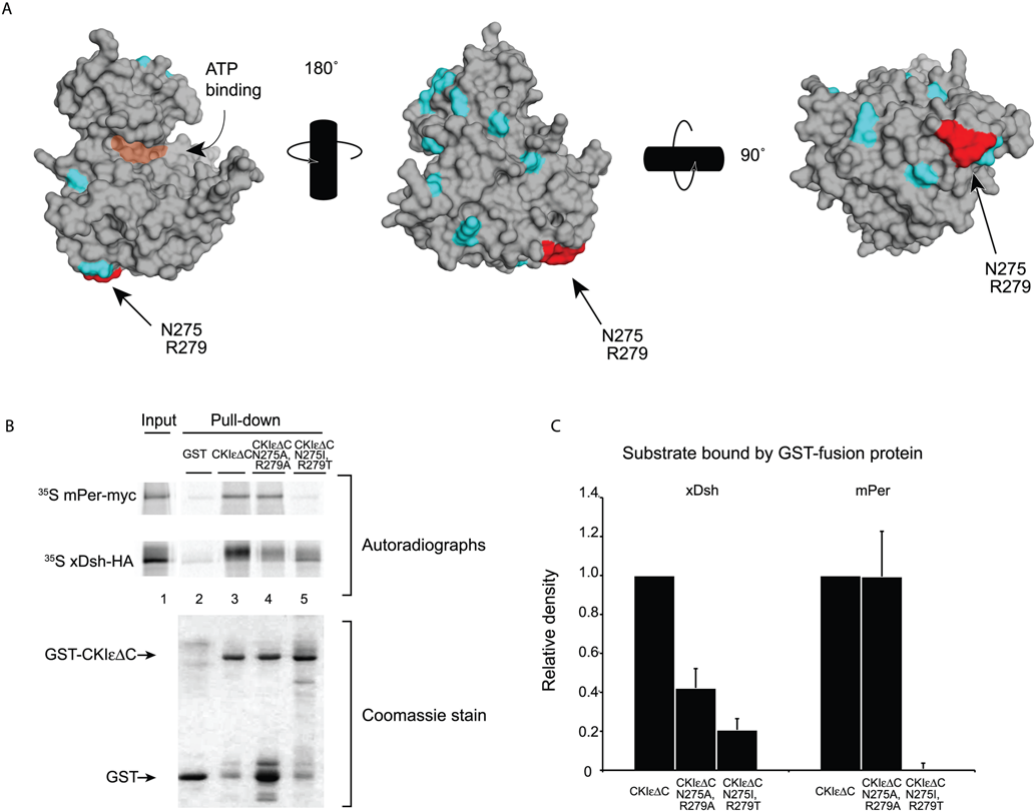
Residues 275 and 279 regulate binding to xDsh and mPer1. (A) Space-filling representation of CKIδ (PDB ID 1CKJ, [50]). Residues shown in cyan and red are conserved between CKIε and CKIδ, but not CKIα. Red residues N275 and R279 are solvent accessible and are chemically distinct in CKIα. Orange shading shows the position of the ATP binding cleft. (B) Binding of ^35^S-labeled mPer1 and xDsh to GST-CKIεΔC, GST-CKIεΔC N275A/R279A, or GST-CKIεΔC N275I/R279T was performed. (C) Quantification of three independent experiments. Values are normalized against the amount of protein bound by GST-CKIεΔC.

To identify residues within the CKIε kinase domain that could confer specificity, we examined the structure of CKIδ. CKIδ is considered a very close relative of CKIε since they share 82% identity over the entire protein and 96% identity in their kinase domains, and since their cellular activities are indistinguishable [11], [13], [24], [48], [52]. Residues shown in cyan in Figure 4A are conserved between CKIε and CKIδ but are not conserved in CKIα. Two residues that are conserved in CKIε and CKIδ but not CKIα, and have solvent exposed side-chains, Asparagine 275 (N275) and Arginine 279 (R279), are shown in red (Fig. 4A). In CKIα, the corresponding residues have chemical properties that differ substantially from the CKIε residues (Asp→Ile and Arg→Thr), and therefore these residues could contribute specificity to CKIε-substrate interactions.

We initially changed N275 and R279 to alanines in the context of the conventional CKIεΔC truncation (CKIεΔC N275A/R279A, Fig. 2). These mutations significantly disrupted the binding of xDsh to CKIε, although they had no effect on mPer1 binding (Fig. 4B, lane 4, and Fig. 4C). However, when we replaced N275 and R279 with the corresponding CKIα residues Ile and Thr, respectively (CKIεΔC N275I/R279T, Fig. 2), the binding between CKIε and mPer1 was abolished and the binding between CKIεΔC and xDsh was further reduced (Fig. 4B, lane 5, and Fig. 4C). In conclusion, N275 and R279 are required for strong binding between CKIε and xDsh, whereas the corresponding chemically distinct residues in CKIα prevent binding of mPer1 to CKIε.

### The C-terminus of CKIε modulates the effect of mutations in the kinase domain

**Figure 5:**
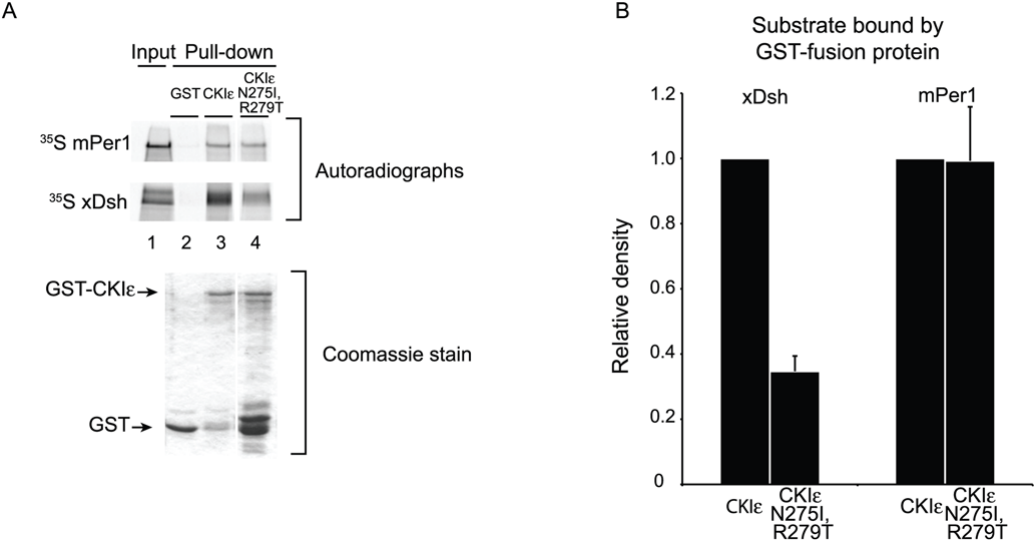
The C-terminal tail enhances binding of xDsh and mPer1 to mutant CKIε. (A) GST, GST-CKIε, or GST-CKIε N275I/R279T was bound to glutathione sepharose and incubated with ^35^S-labeled xDsh or mPer1. (B) Quantification of three independent experiments. Values are normalized against the amount of protein bound by GST-CKIε.

In order to determine what role the C-terminus might play in substrate recognition, we introduced the N275I and R279T substitutions into full-length CKIε (CKIε N275I/R279T, Fig. 2). xDsh's binding to CKIε N275I/R279T is marginally enhanced compared to its binding to CKIεΔC N275I/R279T, while mPer1 binding is completely restored (compare Fig. 4 to Fig. 5). We conclude that the C-terminus is not necessary for CKIε to bind to substrates (see Fig. 3), but that it stabilizes otherwise weakened interactions between xDsh or mPer1 and CKIε.

**Figure 6:**
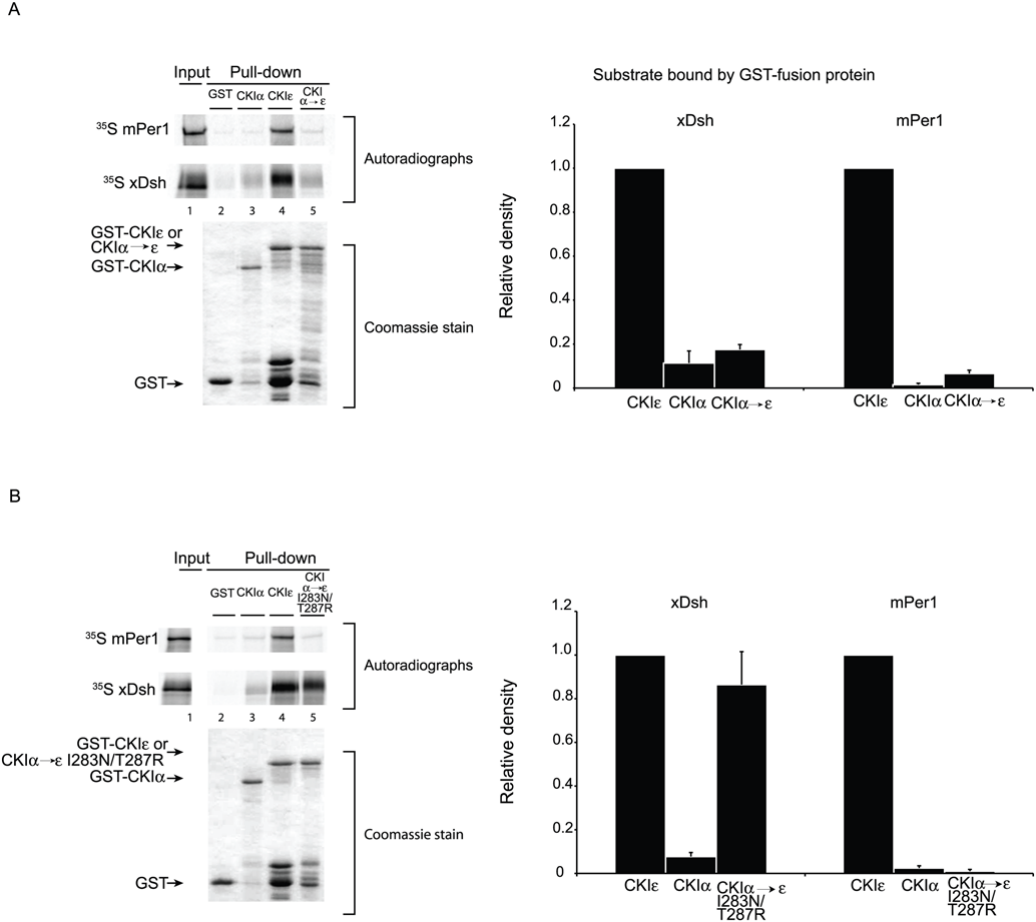
A. Neither xDsh nor mPer1 bind strongly to CKIα→ε. GST, GST-CKIε (partially purified) or GST- CKIα→ε, was bound to glutathione sepharose and incubated with ^35^S-labeled xDsh or mPer1. (B) GST, GST-CKIε, or GST- CKIα→ε I283N/T287R, was bound to glutathione sepharose and incubated with ^35^S-labeled xDsh or mPer1. The bar graphs represent quantification of three independent experiments. Values are normalized against the amount of protein bound to GST-CKIε.

We constructed a chimeric protein that contains the kinase domain of CKIα and the C-terminus of CKIε to determine if the C-terminus of CKIε could confer CKIε-like binding to CKIα (CKIα→ε, Fig. 2). Neither xDsh nor mPer1 showed strong binding to the chimeric protein, compared to CKIε (Fig. 6A). Their weak binding to CKIα→ε along with the data presented above indicates that the C-terminus is able to contact and partially stabilize the interaction between CKIε and its substrates, but it is not sufficient for the binding of mPer1 or xDsh to CKI.

### Conversion of CKIα to an xDsh-binding protein

We hypothesized that CKIα's residues I283 and T287, which correspond to CKIε's N275 and R279, were responsible for preventing interactions with CKIα→ε. We therefore engineered CKIα→ε I283N/T287R, which is nearly identical to CKIα→ε but contains two point mutations that could potentially enhance binding by xDsh and mPer1 (Fig. 2). Impressively, conversion of CKIα residues I283 and T287 to the CKIε identity enabled xDsh to fully interact with the kinase. In contrast, mPer1 did not bind CKIα→ε I283N/T287R. This data clearly demonstrates that xDsh depends on the CKIε residues N275 and R279 for strong binding to CKIε, and reveals that mPer1 requires an additional CKIε-like environment elsewhere on the kinase in order to bind CKIα.

### Phosphorylation of the C-terminus of CKIε inhibits the binding of target proteins

**Figure 7:**
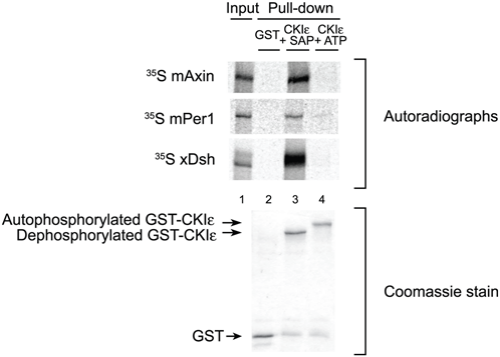
Autophosphorylation of CKIε inhibits binding by substrate and scaffolding proteins. Purified GST and GST-fusion proteins were bound to glutathione sepharose and bound GST-CKIε was incubated with SAP or ATP for 1 hour. Resin was incubated with ^35^S-Methionine-labeled xDsh, mAxin or mPer1. 10% of the mPer1and mAxin input and 25% of the xDsh input were run. Coomassie stained gel shows the levels of GST fusion protein used for each pull-down.

Our data shows that the C-terminus of CKIε stabilizes some substrate interactions, specifically in the absence of motifs that are required for binding (Fig. 6A and B). However, autophosphorylation of the C-terminus has also been shown to inhibit CKIε's activity towards protein targets [6], [7], [53], [54]. We hypothesized that this effect might be partially mediated by a change in affinity for substrates. When CKIε is allowed to fully autophosphorylate, neither mPer1 nor xDsh bind to it (Fig. 7). In addition, hyperphosphorylated CKIε no longer binds to mAxin, a protein that is able to bind to both CKIα and CKIε. Hyperphosphorylation of CKIε's C-terminus thus inhibits the binding of substrate proteins, and may act as a regulatory mechanism to control phosphorylation of targets.

### Autophosphorylation of the C-terminus of CKIε stabilizes its interaction with the kinase domain

Despite numerous attempts, we were unable to crystallize full-length autophosphorylated CKIε, raising the possibility that the phosphorylated C-terminus does not adopt a single conformation, which is necessary to obtain a crystal. It was possible, however, that the autophosphorylated C-terminus of CKIε adopts a stable set of conformations that blocks the binding site for substrates. As an alternative approach to crystallography, we performed a set of crosslinking and mass spectrometry experiments to establish how autophosphorylation of CKIε's C-terminus interferes with substrate binding.

**Figure 8:**
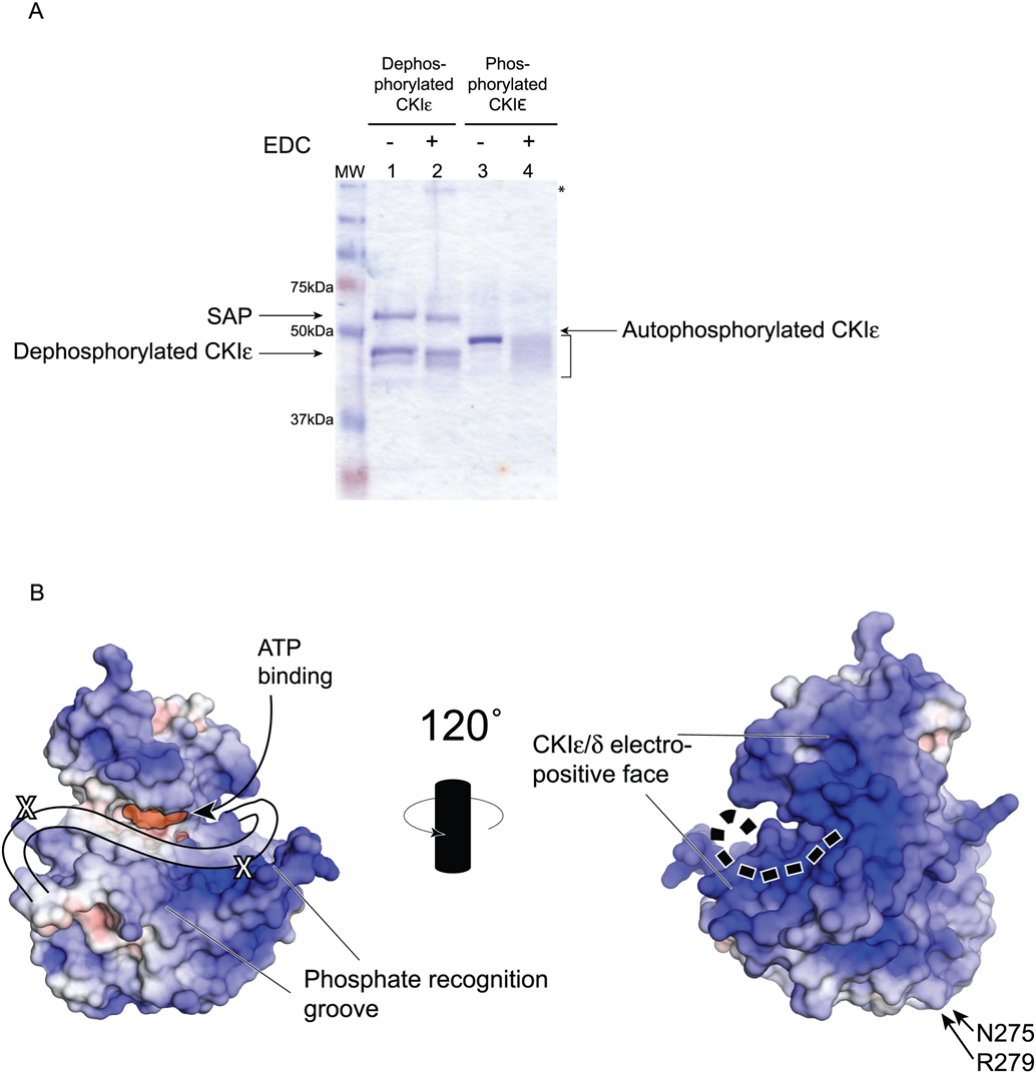
Analysis of the binding of the C-terminal tail. (A) Full-length CKIε was incubated with either SAP or ATP prior to reaction with the EDC crosslinker. Lane 2 shows that there is no change in the apparent molecular weight of dephosphorylated CKIε. In lane 4, there is marked change in the migration of autophosphorylated, crosslinked CKIε (bracketed). Asterisk shows a high molecular weight species that may correspond to SAP-CKIε oligomers (lane 2). (B) Space-filling models of CKIδ are shown. The APBS plugin for PyMol (DeLano Scientific LLC) was used to establish electrostatic potential of solvent exposed atoms. Positively charged areas are shaded blue and correspond to basic regions of the protein; negatively charged regions are red, and correspond to acidic areas. The highly basic groove that has been postulated to be a phosphate recognition region is conservered across the CKI family. The two identified cross-linked residues are indicated with an X. The cartoon line on the left side diagram shows the position of the first 20 amino acids of the tail based on the crosslinking data. The dotted line on the right side shows the proposed extension of the tail onto the backside of the kinase.

We first looked for evidence of a conformational change by following differences in electrophoretic mobility of purified, hyperphosphorylated CKIε upon the addition of 1-Ethyl-3-[3-dimethylaminopropyl]carbodiimide Hydrochloride (EDC), a zero-length crosslinker. When two or more proteins are crosslinked by EDC, the expected product is a band of approximately their additive molecular weights. In the case of CKIε, no high molecular weight bands appear, demonstrating that CKIε does not form dimers or higher-order oligomers in solution. Instead, the single band corresponding to CKIε broadened considerably, and showed faster mobility after EDC treatment as seen by PAGE analysis (Fig. 8A, lane 4). Interestingly, unphosphorylated CKIε did not show a change in mobility after crosslinking and it continued to migrate as a single band (Fig. 8A, lane 2). The broad band that appears after crosslinking suggests that the autophosphorylated CKIε occupies multiple conformations.

We investigated where CKIε's hyperphosphorylated C-terminus binds to the kinase domain using mass spectrometry on three different trypsin-digested preparations of phosphorylated and then crosslinked protein. In order to alleviate potential problems with phosphorylation inhibiting tryptic cleavage or detection of peptides on the mass spectrometer, we treated one sample with Shrimp Alkaline Phosphatase (SAP) after crosslinking but before trypsin cleavage. SAP treatment reduced the level of phosphorylation of crosslinked CKIε as seen by radioactive labeling on the protein (data not shown).

Mass spectrometry analysis showed that the sequence PEDLDRERREHDREER is crosslinked to the kinase domain (Fig. S1 and Fig. S2). PEDLDRERREHDREER corresponds to the 20 amino-acid stretch that was removed from CKIεΔC to make CKIεΔΔC (Fig. 2). Two crosslinks along the PEDLDRERREHDREER peptide position it directly along the basic groove that abuts the active site of CKIε, which was postulated to be a possible phosphate recognition region [50], [55]–[60] (Fig. 8B). Lysines 130 and 232 are part of the kinase domain and were crosslinked to opposite ends of the peptide (P**E**DLD and EHDR**E**ER, respectively). We did not identify any crosslinks in the rest of the C-terminus. This is in agreement with our PAGE results, which indicate that the crosslinked protein sample is highly heterogeneous and would have reduced abundance of unique cross-linked peptides (Fig. 8A).

We also collected spectra for two phosphorylated peptides that were not crosslinked efficiently. These peptides, MGQLRGS(p)AT(p)RALPPGPPAGAAPNR and ISASQAS(p)VPFDHLGK (Fig. S3 and Fig. S4) are phosphorylated at previously described autoinhibitory autophosphorylation sites [6]. The two phosphorylated peptides were found even in the sample treated with SAP. Presumably dephosphorylation of these tryptic peptides was blocked due to inefficient contacts between SAP and the phosphates, suggesting that the phosphate modifications may be protected through interactions with the rest of CKIε.

## Discussion

CKIε and CKIα are closely related kinases with very divergent C-termini. We show here that recombinant CKIε and CKIα interact in vitro with two known CKIε substrates similarly to the previously published in vivo interactions between endogenous proteins [23], [47]–[49]. Using recombinant proteins, we demonstrate that CKIε's C-terminus is not essential for binding to xDsh and mPer1, and show that two residues in the conserved kinase domain, N275 and R279, provide a critical interface for binding by these substrates. Additionally, we show that the C-terminus of CKIε can positively regulate substrates by stabilizing contacts with the kinase domain when dephosphorylated, whereas it can negatively regulate substrate-kinase interactions when autophosphorylated. Finally, we show that a conformational change occurs in the C-terminus of CKIε such that the C-terminus probably binds near the active site when the kinase is autophosphorylated.

### Selectivity of CKIε binding for Dishevelled

**Figure 9:**
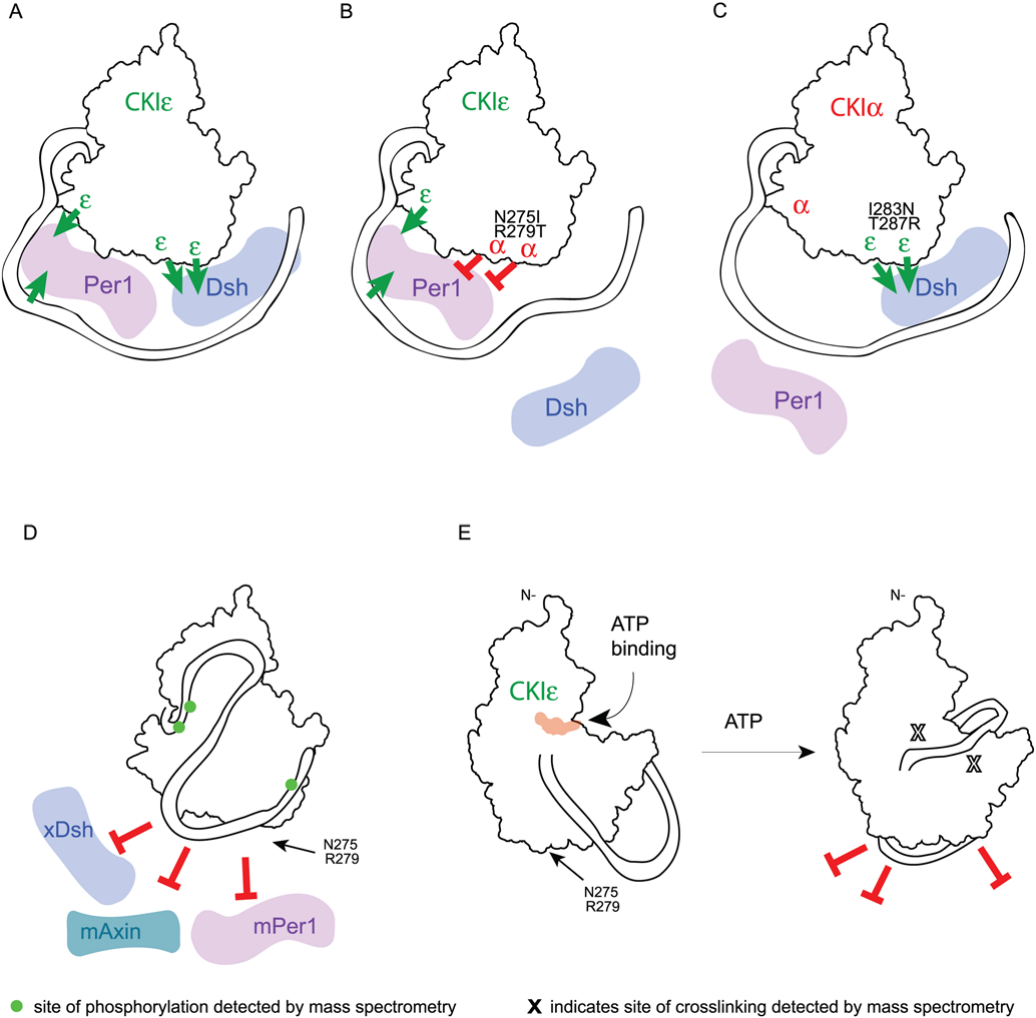
Model of inter- and intra-molecular interactions involving CKIε. Red blocked arrows represent inhibition of binding and green arrows represent positive binding interactions. (A–D) Back face of the kinase. (A) CKIε binds to Dsh and Per using different binding sites. (B) Mutation of CKIε N275 and R279 to the corresponding CKIα identity inhibits Dsh and Per binding; however, the C-terminal tail and at least one other residue in the kinase domain promote Per binding. (C) Changing two residues in CKIα to the CKIε identity along with adding CKIε's C-terminus enables Dsh to bind to CKIα. Per is unable to bind this chimeric kinase. (D) Binding of the autophosphorylated tail to the backside prevents the binding of substrates. Phosphorylated sites detected by mass spectrometry are shown on the tail. (E) View of the front side of the kinase. Left, CKIε's C-terminus is labile when it is not phosphorylated, and CKIε is able to bind to partners. Right, upon incubation with ATP, CKIε autophosphorylates and the C-terminus binds tightly to the back side of the kinase domain. This positions the peptide PEDLDRERREHDREER next to the active site and the phosphate recognition groove. The X's show identified crosslinks between the peptide and the kinase domain.

The binding of xDsh to CKIε and not CKIα primarily requires the residues N275 and R279 (Fig. 9A). Mutation of these residues to either alanines or the CKIα identities, isoleucine and threonine respectively (Fig. 9B), prevented binding. Thus, the identity of these residues is essential, suggesting that Dsh directly contacts these residues. We also noted that the extent to which Dsh was phosphorylated (visualized by a change in electrophoretic mobility, Fig. 4) was different depending on the mutations that were made. The identities of the residues at positions 275 and 279 in CKIε may therefore also be important for correctly positioning Dsh for full phosphorylation. The C-terminal tail of CKIε provided some enhancement of xDsh binding and increased the level of phosphorylation by the mutant protein (Fig. 5), but it was not sufficient for binding. Strikingly, we saw full binding of xDsh to CKIα simply by changing the I283 and T287 to the CKIε identity and adding on the C-terminal tail (Fig. 9C). In the absence of the C-terminal tail, Dsh did not bind to the mutated CKIα (data not shown), which strengthens our hypothesis that the C-terminus of CKIε does contact Dsh, but is not required in for interactions with wild-type CKIε. These results allow us to fully explain the selectivity of CKIε for Dishevelled.

### Selectivity of CKIε binding for Period

The binding of mPer1 to CKIε is more complex than for Dishevelled. Changing residues N275 and R279 to alanine in the context of CKIεΔC did not disrupt mPer1 binding, whereas mutation of these residues to the CKIα identity in CKIεΔC eliminated all binding. This result indicates that the CKIα residues at this position prevent binding by mPer1, but that the residues may not be directly involved in its binding (Fig. 9B). This is supported by the observation that changing these residues in CKIα to the CKIε identity (as well as adding the C-terminal tail) did not rescue mPer1 binding even though it completely rescued xDsh binding.

The C-terminal tail does have an important contribution to mPer1 binding since full-length CKIε N275I/R279T could fully bind mPer1 whereas CKIεΔC N275I/R279T could not bind mPer1. Thus the C-terminal tail can override the repulsive effect of N275I and R279T. However, since the tail did not rescue CKIα→ε I283N/T287R, it alone is not sufficient for binding mPer1 to CKIα. These results reveal that another site (or sites) on CKI must also contribute to the difference in binding between CKIε and CKIα.

One CKI-binding motif, F-X-X-X-F (where F is phenylalanine and X can be any amino acid residue), is present in mPer1 but not in xDsh [49]. Studies of the NFAT family of proteins suggest that the F-X-X-X-F motif enables stable interactions with both CKIα and CKIε [49]. Mutation of the phenylalanine residues to alanines abolishes binding by NFAT to CKIα and CKIε. mPer1 shows the same loss of binding when its F-X-X-X-F motif is mutated [37], [49]. Since the F-X-X-X-F motif binds both CKIα and CKIε, it contributes to the binding affinity of mPer1 to CK1 but it is not involved in the selectivity. Thus, we conclude that another residue (or residues) on CKIε besides N275 and R279 contributes to the selectivity (Fig. 9A). This additional residue (or residues) could be amino acids that in CKIε positively enhance the binding of mPer1, or residue (or residues) that in CKIα repel mPer1. Nonetheless, our results demonstrate that the lack of the tail and changes in two residues in CKIα are sufficient to explain the lack of binding of CKIα to mPer1.

### The role and structure of the C-terminal tail

The C-terminus of CKIε has been shown in previous reports to be catalytically autoinhibitory and important for regulation within signaling pathways [61]–[63]. Wnt signaling lowers the level of autophosphorylation on CKIε's C-terminus [63] though only a subset of the sites are phosphorylated in vivo, judging by polyacrylamide gel and isoelectric focusing data. Our data show that phosphorylation of the C-terminus blocks interactions between CKIε and binding partners, and it is therefore likely that some portion of the lowered phosphorylation activity seen in vivo is due to less efficient substrate binding.

The C-terminus of CKIε has been proposed to act as a pseudo-substrate for the kinase during autoinhibition [4], [50], [57]. The positively charged groove in front of the active site has been postulated to bind phosphopeptides that helps determine the S(p)-X-X-S specificity of the kinase family [1], [4], [50], [55]–[59], [64]. Thus the phosphates in the tail could occupy that site and prevent substrates from binding. Our data suggest the alternative view that the phosphorylation of the tail causes the unphosphorylated but highly charged peptide, PEDLDRERREHDREER to sit across the front of the kinase's active site when the C-terminal tail is autophosphorylated, thus preventing substrate access to the active site and/or phosphate recognition groove (Fig. 8B and 9E). Intriguingly, two previous structures of CKIδΔC failed to show any density for this peptide [50], [58], indicating that phosphorylation of the C-terminal tail is necessary to lock this peptide into place.

In the model illustrated in Figure 9D, after PEDLDRERREHDREER is directed through the phosphate recognition groove, the rest of the C-terminus is led to the back of the kinase (Fig. 9D). The back face is very basic as shown in an electrostatic view (Fig. 8B), providing many sites for the phosphates in the tail to interact. The heterogeneity of the cross-linked phosphorylated CKIε and the lack of identification of prominent cross-linked tail peptides suggests that the interaction of the tail with the back side is highly variable, which also explains our inability to crystallize full-length CKIε (the same was also found with full-length CKIδ [50], [58]). Once at the back side of the kinase domain, some phosphorylated residues on the C-terminus are buried in the basic face to stabilize the conformation (Fig. 9E). The long C-terminus could then fold against the back side of the kinase domain to occlude the binding sites for Dsh, Per and Axin. Thus, we suggest that phosphorylation of the tail has two roles, to bring the proximal C-terminus in front of the active site to prevent substrate phosphorylation and to prevent substrate binding using the distal part of the tail.

Casein Kinase family members have many targets in the cell. The results of this study designate two amino acid residues that determine the ability of two different CKI isoforms to select and bind their target proteins. Furthermore, our work on auto- and dephosphorylated CKIε introduces a means to regulate CKIε's in the presence of its targets. These two biochemical mechanisms could help maintain the strict spatial and temporal control of CKIε and CKIα that is necessary in both the circadian rhythms and Wnt pathways.

## Materials and Methods

### Expression of GST-CKIε, GST-CKIα and CKI mutant proteins

GST-CKIε was expressed in *E. coli* in Terrific Broth for 18 hours at 16°C. Cells were grown to OD_600_ = 2 in baffled flasks and were induced with 0.5 mM IPTG. Cells were pelleted and opened by sonication, in the presence of PMSF, Leupeptin and Pepstatin A. Lysate was cleared by centrifugation, and was applied to Glutathione Sepharose 4B resin (Amersham) or Glutathione Sepharose resin (Clontech). Resin was washed three times with 20 mM Tris, pH 8, 250 mM NaCl, 5 mM DTT and the GST fusion protein was eluted with 15 mM glutathione in the same buffer. Protein was dialyzed overnight against 20 mM Tris, pH 8, 50 mM NaCl, 5 mM DTT at 4°C and was applied to 1 or 5 mL HiTrap Q sepharose column (Amersham, GE Healthcare). Protein was eluted by ion exchange using 20 mM Tris, pH 8, 800 mM NaCl, 1 mM DTT. Fractions containing the cleanest fusion protein were frozen in liquid nitrogen and stored at −80°C. For generation of CKIε without the GST-tag, ion-exchange fractions were pooled and re-bound to glutathione resin. After being washed with 20 mM Tris, pH 8, 250 mM NaCl, 5 mM DTT, the resin was incubated over night with TEV protease at room temperature, in the same buffer, with 1 mM EDTA and 1 mM DTT added. The supernatant from the overnight cleavage contained CKIε, which was either frozen in liquid nitrogen and stored at −80° C, or was further purified by gel filtration on a Sephadex 200SE column. Gel filtration fractions were pooled and concentrated on VivaSpin MWCO 10,000 filtration devices (GE Healthcare) and were frozen in liquid nitrogen and stored at −80°C.

GST-CKIα was expressed in *E. coli*, in Luria Broth for three hours at 18°C. Cells were grown to OD_600_ = 1, and were induced with 0.5 mM IPTG. Mutants of CKIα and CKIε were expressed at 18–24°C, for 3–18 hours, depending on the construct. Cells were grown to OD_600_ = 1, and were induced with 0.5 mM IPTG.

GST was expressed in *E. coli* from the pGex-4T1 plasmid as in [65].

### Plasmids and GST-CKIε mutants

mPer1-myc was a generous gift from D. Virshup. xDsh-HA is as in [22]. mAxin-myc is from [66]. XCKIε -GST was created by inserting CKIε (EST) from *Xenopus laevis* into pGEX-4T1. XCKIα-GST was made by inserting CKIα (EST) from *Xenopus laevis* into pGEX-4T1. Mutagenesis of CKIα and CKIε was performed according the Stratagene QuikChange protocol. GST-CKIα−>ε -tail was constructed such that the CKIα sequence ends at residue 296, and the CKIε sequence begins with CKIε residue 288.

### GST pull-down experiments

Pull-down experiments were as performed in [65] with the following differences. For most experiments, cell pellets were thawed, sonicated and the lysate was applied to glutathione resin for 10 minutes at room temperature. Beads were washed three times with wash buffer (20 mM Tris, pH 7.5, 150 mM NaCl, 0.5% Nonidet P-40, 1 mM DTT). Binding reactions were done at 4° C for 40 minutes in wash buffer. After binding, beads were washed three times for 5–10 minutes with wash buffer prior to addition of sample buffer. Except where noted, all full-length CKIε and CKIα→ε constructs were dephosphorylated using Shrimp Alkaline Phosphatase (Fermentas) for 1.5 hours at 30°C after binding to glutathione sepharose. The bound protein was then washed three times to remove traces of phosphatase. For experiments requiring hyperphosphorylated CKIε, GST-CKIε was bound to glutathione and allowed to autophosphorylate in the presence of 10 mM MgCl_2_ and 2 mM ATP for 1.5 hours. Except where noted, all experiments that required full-length CKIε were run with chromatographically purified protein (including crosslinking and mass spectrometry experiments) and all other experiments were run with partially purified protein from crude *E. coli* lysate.

### Crosslinking and mass spectrometry

Purified CKIε (2 mg/mL) was incubated with 10 mM MgCl_2_ and 10 mM ATP for 1.5 hours, at 30°C. CKIε was diluted 4-fold in 100 mM potassium phosphate buffer (pH 7.5) and incubated with 100 mM EDC (Pierce) for three hours at room temperature. The crosslinked protein was dialyzed against two changes of 20 mM Tris, pH 7.5, 50 mM NaCl for 5 hours at 4°C. Protein was concentrated by evaporation to 10 µL and resuspended in 25 mM Ammonium bicarbonate (pH 7.8) in 6 M Urea. CKIε that was dephosphorylated prior to trypsinization was incubated with SAP for 1.5 hours at 30°C after dialysis; tryptic peptides that were dephosphorylated were also incubated with SAP for 1.5 hours at 30°C, immediately after tryptic digestion. Peptides were desalted on C-18 spin columns (The Net Group, Inc) and infused into a hybrid-LTQ-Orbitrap mass spectrometer via electrospray ionization (ESI). Crosslinked peptides were identified by using the open modification search tool POPITAM [67]. Post-translational modifications that corresponded to peptides were searched to identify intramolecular crosslinks.

## Supporting Information

Figure S1 Tandem mass spectrum of cross-linked peptide from dephosphorylated CKIε with a precursor mass of 2686.3876 daltons. All labeled fragment ions were identified with more than 10 ppm mass accuracy, written in red above the ion peak. Ions that are labeled in green are from peptide DVKPDNILMGLGKK and ions labeled in blue are from peptide NPEDLDRER. Solid horizontal bars mark ions whose relative abundance was greater than the scale at left.
(1.02 MB EPS)

Figure S2 Tandem mass spectrum of cross-linked peptide from phosphorylated CKIε with a precursor mass of 3982.0332 daltons. All labeled fragment ions were identified with more than 10 ppm mass accuracy, written in red above the ion peak. Ions labeled in green are from peptide KMSTPIEVLCK and ions labeled in blue are from peptide FGAARNPEDLDRERREHDREER. Solid horizontal bars mark ions whose relative abundance was greater than the scale at left.
(0.73 MB EPS)

Figure S3 Tandem mass spectrum of phosphorylated peptide MGQLRGSATRALPPGPPAGAAPNR with a precursor mass of 2502.7794 daltons. All labeled fragments were identified with SEQUEST, and the spectrum shown was taken directly from the SEQUEST results. Charge states are indicated by +, and phosphorylated residues are represented by lower case letters in the peptide sequence.
(0.62 MB EPS)

Figure S4 Tandem mass spectrum of phosphorylated peptide ISASQASVPFDHLGK with precursor mass of 1636.5736 daltons. All labeled fragments identified with SEQUEST, and the spectrum shown was taken directly from the SEQUEST results. Charge states are indicated by +, and phosphorylated residues are represented by lower case letters in the peptide sequence.
(0.60 MB EPS)
